# Crop DNA extraction with lab-made magnetic nanoparticles

**DOI:** 10.1371/journal.pone.0296847

**Published:** 2024-01-08

**Authors:** Haichuan Wang, Xueqi Zhao, Li Tan, Junwei Zhu, David Hyten

**Affiliations:** 1 Department of Agronomy and Horticulture, University of Nebraska-Lincoln, Lincoln, Nebraska, United States of America; 2 Department of Mechanical & Materials Engineering, University of Nebraska-Lincoln, Lincoln, Nebraska, United States of America; 3 MOE Key Laboratory for Nonequilibrium Synthesis and Modulation of Condensed Matter, School of Science, Xi’an Jiaotong University, Xi’an, China; 4 USDA-ARS, Lincoln, Nebraska, United States of America; Hainan University, CHINA

## Abstract

Molecular breeding methods, such as marker-assisted selection and genomic selection, require high-throughput and cost-effective methods for isolating genomic DNA from plants, specifically from crop tissue or seed with high polysaccharides, lipids, and proteins. A quick and inexpensive high-throughput method for isolating genomic DNA from seed and leaf tissue from multiple crops was tested with a DNA isolation method that combines CTAB extraction buffer and lab-made SA-coated magnetic nanoparticles. This method is capable of isolating quality genomic DNA from leaf tissue and seeds in less than 2 hours with fewer steps than a standard CTAB extraction method. The yield of the genomic DNA was 582–729 ng per 5 leaf discs or 216–1869 ng per seed in soybean, 2.92–62.6 ng per 5 leaf discs or 78.9–219 ng per seed in wheat, and 30.9–35.4 ng per 5 leaf discs in maize. The isolated DNA was tested with multiple molecular breeding methods and was found to be of sufficient quality and quantity for PCR and targeted genotyping by sequencing methods such as molecular inversion probes (MIPs). The combination of SA-coated magnetic nanoparticles and CTAB extraction buffer is a fast, simple, and environmentally friendly, high-throughput method for both leaf tissues and seed(s) DNA preparation at low cost per sample. The DNA obtained from this method can be deployed in applied breeding programs for marker-assisted selection or genomic selection.

## Introduction

High-quality DNA is essential for experiments involving DNA manipulation for its wide range of applications in molecular breeding and research. The development of new technologies, such as second generation sequencing, third-generation long-read sequencing technology [1], and targeted sequencing such as molecular inversion probe (MIP) [2–4] have enabled high-throughput genotyping and sequencing of crops in applied breeding programs. To enable the analysis of the number of lines analyzed in breeding programs, a high-throughput, quick and efficient method is needed for the isolation of high-quality genomic DNA.

Many plant DNA extraction protocols comprise of the disruption of the cell to release the DNA into solution. This is followed by the precipitation of DNA to enable the removal of the unrelated biomolecules such as the proteins, polysaccharides, lipids and other secondary metabolites. Since DNA can be extracted from various types of tissues such as leaves, cotyledons, seeds, endosperm, tissue culture callus, roots etc., the tissue type along with the concentration and quality of the DNA required by the researcher helps determine the DNA extraction protocol needed to be employed. A commonly used basic plant DNA extraction protocol when high quality DNA is needed are modified versions of the protocol from Murray and Thompson [5] and Saghai-Maroof et al. [6]. These modifications help adapt the protocol to fit a particular plant and tissue type based on the cell walls and cellular components. Plants have leaves with major sclerenchyma tissues and high seed protein and/or starch content [7–9]. This can limit the quality and quantity of DNA obtained from DNA extraction protocols. There are modified CTAB methods to isolate genomic DNA from leaf and seed of cereal crops that overcome these challenges and do provide high quality DNA suitable for most molecular breeding methods [10]. However, some organic compounds, such as phenol and chloroform are major components in these DNA extraction protocols and can cause health issues (such as burning to the eyes and skin, eye irritant, and carcinogen and reproductive hazard) to humans (https://ehrs.upenn.edu/health-safety/lab-safety/chemical-hygiene-plan/fact-sheets/fact-sheet-phenol-chloroform-extraction). In addition to modified CTAB methods, there are column-based nucleic acid extraction kits available for crops that provide high quality DNA, however, these kits can be costly to deploy for molecular breeding methods being applied to thousands of samples at a time.

Magnetic nanomaterials (nanoparticles) provide solid phase for the enrichment and purification of biomolecules directly from chemical or biological suspensions. Recently, the application of magnetic nanoparticles has attracted attention for their use in isolating DNA and RNA from a variety of materials, such as cell culture [11], bacterial, animal, and plant [12, 13]. Salicylic acid (SA) has strong affinity to nucleic acids, the attachment of the salicylate entities (SA) to Fe_3_O_4_ surface will allow the nucleic acid (such as DNA) attached to SA to be co-isolated with the magnetic nanoparticles when exposed to the magnetic field. Using these nanoparticles to purify DNA has been shown to be highly desirable in terms of cost and efficiency. However, less information is available for its application of providing high quality DNA for molecular breeding methods in major crops such as maize, wheat and soybean.

In this study, we combined the lysis step in CTAB DNA isolation protocol with lab-made nanoparticles for purification for its application to 1) extract high quality DNA from crop leaf tissue and seeds, 2) and test the isolated DNA in downstream molecular breeding methods such as PCR and a targeted sequencing method.

## Material and methods

Iron(II) chloride tetrahydrate (FeCl_2_·4H_2_O) and iron(III) chloride hexahydrate (FeCl_3_·6H_2_O) were obtained from Sigma-Aldrich (USA). Sodium hydroxide (NaOH) and Salicylic acid (SA) were purchased from Amresco (USA) and Alfa Aesar (USA) respectively. All materials were used as received and all solutions were prepared with deionized water accordingly.

### Synthesis of magnetic nanoparticles

Magnetic nanoparticles (Fe_3_O_4_) were synthesized by following the protocol as previously described by Unal [14] and Zhou [11] with slight modifications. In brief, a coprecipitation method using Fe^2+^ and Fe^3+^ ions in basic medium. FeCl_2_·4H_2_O (4.34 g, 0.0218 mol) and FeCl_3_·6H_2_O (10.84 g, 0.04 mol) (the molar ratio of Fe(II) to Fe(III) is 0.55: 1) were dissolved in 94 ml deionized water together, then adequately filtered to remove impurities. NaOH (5.0 M, 50 mL) was added into the solution to obtain black magnetic nanoparticles (pH = 14). Then the solution was heated at 60°C for 30 minutes with vigorous stirring followed immediately by slow stirring for 1 h at 80°C under a nitrogen atmosphere, which eventually resulted in a ferrofluid containing Fe_3_O_4_ nanoparticles. To coat the magnetic particles with SA, 5 g, (0.08 mole) of SA powder was added to the suspension (150 mL) of magnetic nanoparticles at a molar ratio of 0.55 Fe(II): 1 Fe(III): 2 SA, and stirred consistently at 90°C for 4 h. The final product (Fe_3_O_4_ @ SA) was separated from the aqueous solution by using magnetic separation and further washed with pure water until a neutral pH was reached. The final solution with SA coated magnetic particles was kept at room temperature for future application in DNA preparation.

### Plant tissues/seeds collection

Leaf discs in ¼ inch were collected from soybean at V2 stage, wheat at Feekes 2 stage and maize at V3 stage by using a paper puncher (Amazon, A7074005S) S1 Fig. For leaf samples, 5, 10 and 15 leaf discs per collection for each crop in three reps. All leaf discs were lyophilized and stored at room temperature for future application.

For seed samples, 1, 2 and 3 dry wheat seed(s) and 1 dry soybean seed was used for DNA extraction. Three replicates for wheat seeds and replicates for soybean seed.

### DNA extraction

DNA extraction for control DNA from soybean leaf discs was conducted by following CTAB method as described in previous publications [13, 15]. For all other DNA isolation with beads, 500 μl of DNA extraction buffer (Tris-HCl 50 mM, pH 6.5, CTAB 1%, EDTA 10 mM, beta-mercaptoethanol 5%, 1.4 M NaCl and 2% PVP-40) was added into each tube with grinded soybean leaf tissue, then incubated at 65°C for 60 min followed by centrifuging at 3700 rpm for 15 min. For seed(s), 500 μl of DNA extraction buffer were added and incubated overnight at 4°C before grinded. 100 μl supernatant was transferred into a new tube with 2 μl beads (60 ng/μl) and 1 μl of RNase A (1 μg/μl), mixed and left on bench for 10 min at room temperature. To denature the DNA, 100 μl isopropanol was added into each well, mixed and kept on a magnet for 5 min after 10 min incubation on bench at room temperature. The solution was carefully removed, then the beads were washed by adding 100 μl 80% EtOH for 30 sec without disturbing the beads. Ethanal was removed and left to air dry for 5 min. Then 50 μl 10 mM Tris-HCl (pH 8) was added and mixed with a pipette or slightly vortexed. The sample was then incubated at room temperature (not on magnet) for 10 min, placed back on the magnet for 3–5 min. The solution was then transferred with DNA into a new tube.

### DNA quantification and qualification

The quantity of DNA samples was evaluated using Qubit (2.0) by following kit instructions. Meanwhile, 1 μl of DNA/sample was used to evaluate DNA quality on 1.5% agarose gel. Finally, a daughter plate was created and standardized to a final concentration of 12 ng/μl/DNA sample for further evaluation by molecular inversion probe (MIP) and conventional PCR analysis, respectively. All DNA samples were stored in -20°C freezer for further evaluation.

### DNA evaluation by PCR and MIP

PCR primers specific to each crop (S1 Table) were used in downstream analysis with DNA obtained with nanoparticles beads. PCR amplification per well (sample) was performed by using 5 μl of 2x master mix (BioRad, Cat. 172–5310), 0.25 μl of 10 μM indexed primers (Forward and Revers primer respectively) and 1 μl genomic DNA, the final reaction volume was 10 μl/sample. The custom indexed primers used in MIP protocol are available in S3 Table. The PCR was carried out with following conditions in 200 μl PCR tubes (Axygen, Cat. PCR02C), 98°C/30 s, 21x (98°C/10 s, 60°C/30 s, 72°C/30 s), 72°C/60 s, holding at 12°C. The PCR products were visualized on 1.5% agarose gel along with 100–1500 bp DNA ladder.

With soybean DNA samples, an additional test was conducted for its application with the targeted sequencing method, molecular inversion probe (MIP), which has been used for a wide variety of clinical applications [16], and adapted for soybean genotyping [4]. The MIPs reactions were carried out to verify the quality of bead-extracted DNAs as described in a previous publication [4] at 12.5 μl per reaction per sample. In brief, DNA from 10 soybean leaf discs was used as template at 63 ng DNA/reaction/sample. The purified pools of MIP libraries were quantified with the Illumina lib quantification kit (KAPA, KK4824) on a QuantStudio 6 Flex system (Thermo Fisher Scientific) followed by the sequencing on NextSeq 550/500 system (Illumina) at 150 bp single read with customer specific sequencing primers (S2 Table). Sequencing reads were deposited in NCBI BioSample database accession SAMN38842718. The downstream sequencing data analysis with relative software packages was performed as described by Wang et al., (2022).

The protocols described in this peer-reviewed article are published on protocols.io (https://dx.doi.org/10.17504/protocols.io.e6nvwdqm7lmk/v1) and is included for printing purposes as S1 File.

### Statistical analysis

All statistical analysis was performed with a student T-test or one way ANOVA (Tukey HSD) at p<0.05.

## Results

### Synthesis of SA-coated nanoparticles and morphology

**Fig 1:**
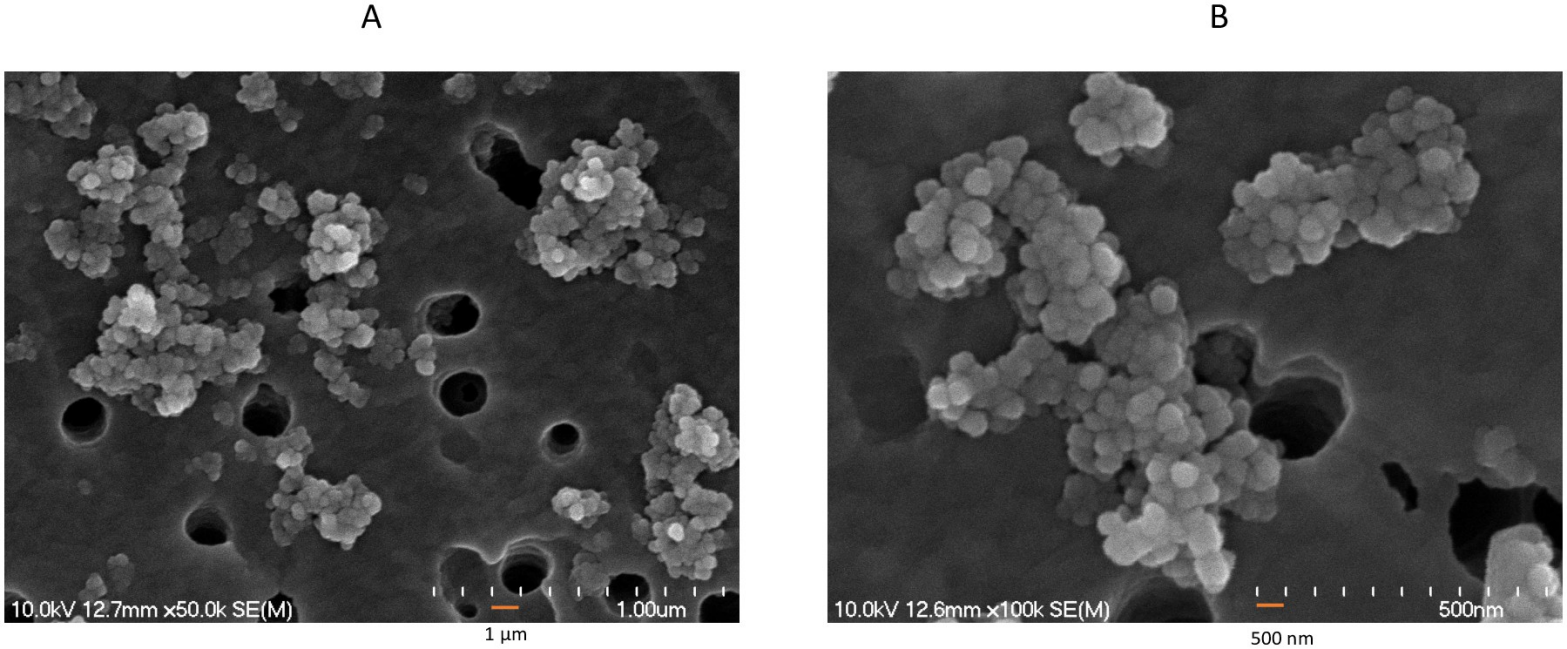
Morphological view of SA-coated nanoparticles under a scanning electron microscope. (A) Scanning electron microscope resolution of 1 μm and (B) 500 nm.

Magnetic nanoparticles (Fe_3_O_4_) were synthesized by a coprecipitation method using Fe^2+^ and Fe^3+^ ions in a basic medium. FeCl_2_·4H_2_O and FeCl_3_·6H_2_O followed by SA coating. Under a scanning electron microscope, the coated magnetic nanoparticles are spherical and nanometric in size at approximately 499 nm±7.4 in diameter (Fig 1).

This is approximately 50x larger than compared to the size of SA-coated nanoparticles (10 nm) produced with original protocol [11]. In addition, the obtained nanocomposites in our experiment were aggregated to a certain level and displayed a variety of morphologies (Fig 1).

In our test, a small scale of each component in weight (gram) were used and produced up to 4 grams of SA-coated magnetic nanoparticles (Table 1).

**Table 1:** Synthesis efficiency of SA-coated magnetic nanoparticles and cost per DNA.

Item	Stock (g)	Price ($)	$/g	Weight (g)	Cost ($)
FeCl_2_.4H_2_O	50	50.5	1.01	4.34	4.38
FeCl_3_.6H_2_O	100	48.62	0.486	10.84	5.27
NaOH	25	40.1	1.604	10.00	16.04
Salicylic acid	100	71	0.71	5.00	3.55
SA coated nanoparticles				4	29.24
1 gram nanoparticle					7.31
120 ng nanoparticles/sample					8.77E-07

Notably, the synthesis of nanoparticles is not limited to certain scale, researchers can change scale proportionally based on their need in experiments or researchers can make more at one time, then share it with other labs or collaborators to make the DNA isolation protocol consistent throughout the entire research project.

### DNA isolation with SA coated magnetic nanoparticles

**Fig 2:**
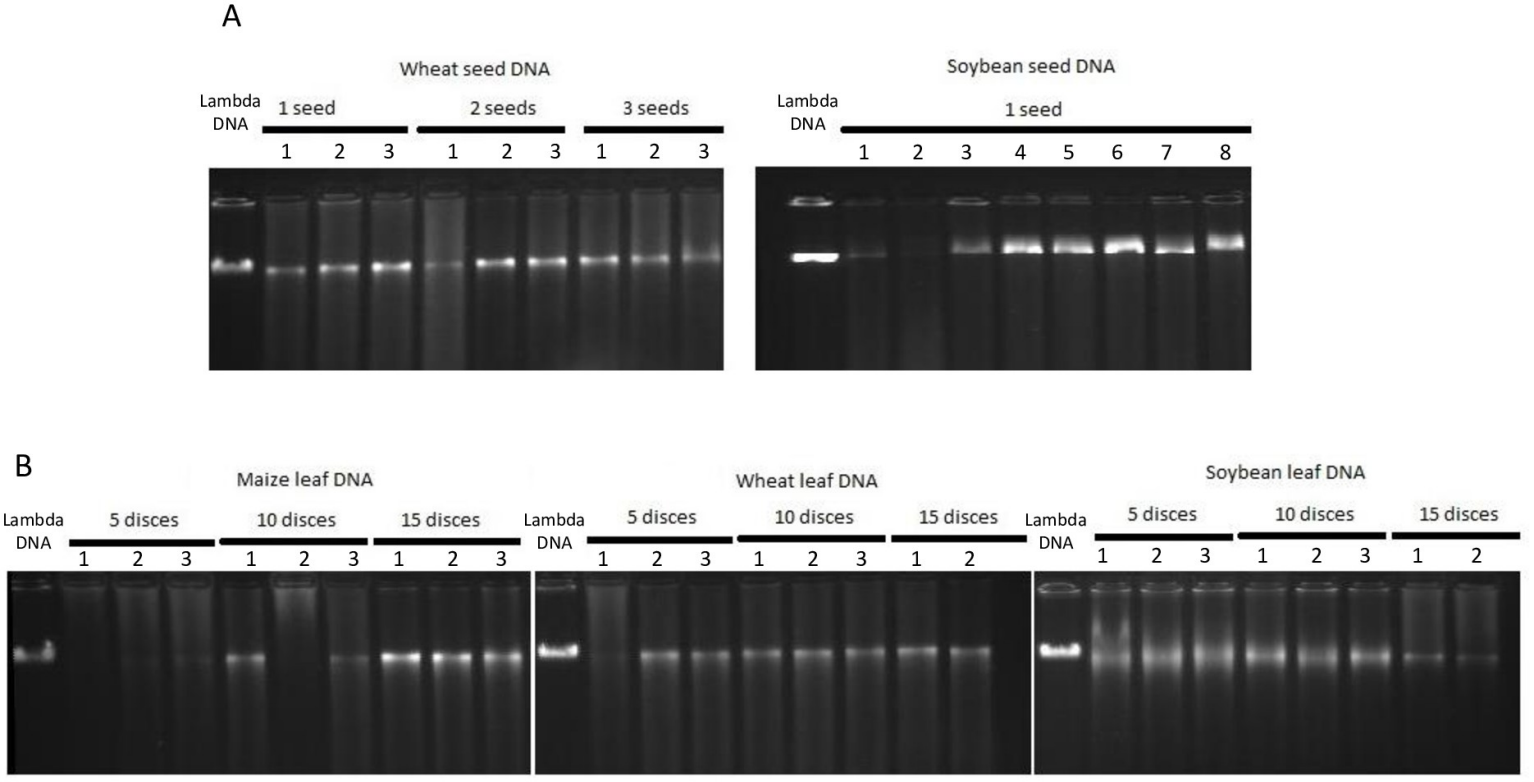
DNA isolated with magnetic beads. Genomic DNA isolated with magnetic beads were run on a 1.5% agarose gel using lambda DNA as a marker. (A) DNA isolated from seed(s) of wheat and soybean and (B) DNA isolated from leaf discs of wheat, soybean and maize.

With SA coated nanoparticles synthesized above, an empirical amount of nanoparticles were used at 120 ng per extraction in DNA preparation from wheat and soybean seeds and leaf discs and maize leaf discs. The DNA prepared with magnetic nanoparticles were evaluated for its quality using 1.5% agarose gel. The data showed that the DNA obtained from the nanoparticles was consistently high molecular weight DNA from both seed(s) (Fig 2a) and leaf tissues (Fig 2b).

There were very few samples that did not have a strong DNA band such as the No. 2 sample in soybean seed or the No.1 sample in wheat leaf (Fig 2). However, there was still evidence of DNA at a detectable level in those samples. For example, 2.92 ng was extracted from the No.1 wheat leaf sample.

The amount of DNA per sample per extraction varied depending on the crop. On average, 127.4±64.8 ng DNA per wheat seed and 1209±624.29 ng DNA per soybean seed were obtained (Table 2, S4 Table).

**Table 2:** DNA yield per seed (s) extraction.

Soybean seed			Wheat seed		
Seed per extraction	Weight (g)	Avg DNA (ng)	Seed (s) per extraction	Weight (g)	Avg DNA (ng)
1	0.1571±0.024	1209±624.29	1	0.0358±0.004	127.4±64.8
			2	0.0638±0.007	297±82.8
			3	0.1235±0.017	1106±211.8

Standard error for three biological replications are provided for each measurement.

The variation of DNA concentration obtained per seed was large, ranging from 216–1869 ng in soybean and 78.9–219 ng in wheat. This variation might be related to the completion of homogenization of seed even though the breakdown of seed was operated with same setting in the Tissue analyzer. DNA from leaf tissue, in the 5-discs category alone, had approximately 677±67.27, 40.6±26.77 and 48.8±22.21 ng DNA obtained from soybean, wheat and maize, respectively (Table 3, S5 Table). The DNA yield from soybean was much higher than that from wheat and maize (p<0.05).

**Table 3:** DNA isolated from leaf discs from three crops.

No of leaf discs	Avg DNA (ng)		
	Soybean	Wheat	Maize
5	677±67.27	40.6±26.77	48.8±22.2
10	855±97.88	90.3±29.58	392.3±68.9
15	1482±96.37	114.0±0.00	1756±219.9

Standard error for three biological replications are provided for each measurement.

The amount of total DNA from leaf was positively correlated with the number of leaf discs used (S2 Fig) in each crop (R^2^ = 0.86, 0.95 and 0.84 for soybean, wheat and maize, respectively)(S5 Table). It is also apparent that much less DNA (except for wheat and maize in 5 leaf discs category) was isolated from wheat leaf tissue in all three-leaf disc category, which is approximately 10 times less as compared to its counterpart in soybean and maize.

### DNA evaluation with PCR

**Fig 3:**
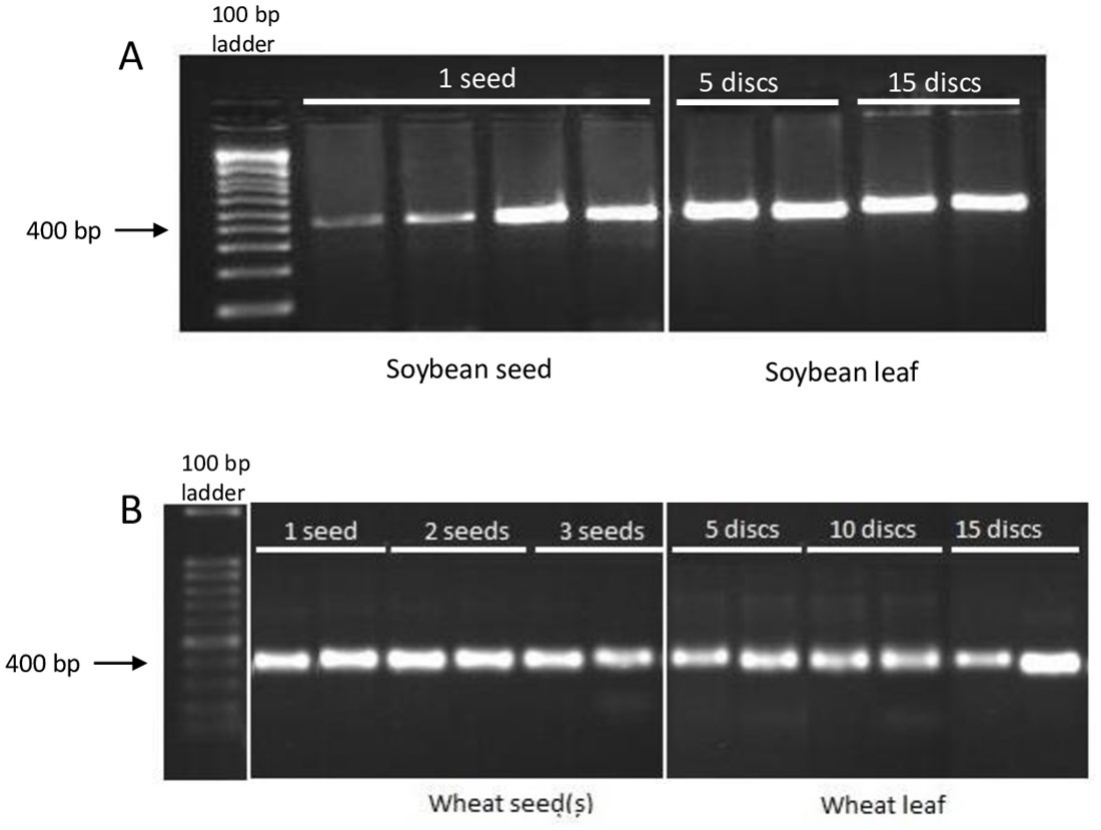
PCR amplification using primer pairs for each crop. (A) PCR with DNA from soybean seed and leaf tissues. (B) PCR products using DNA from maize leaf, wheat leaf and wheat seeds.

The isolated DNA was used as a template for randomly selected DNA samples to assess how well it works for PCR. All PCR reactions generated the desired PCR product at the expected size with DNA from all three crops (Fig 3). No difference of PCR performance was observed between leaf DNA and seed DNA in wheat and soybean.

### DNA evaluation with MIP

**Fig 4:**
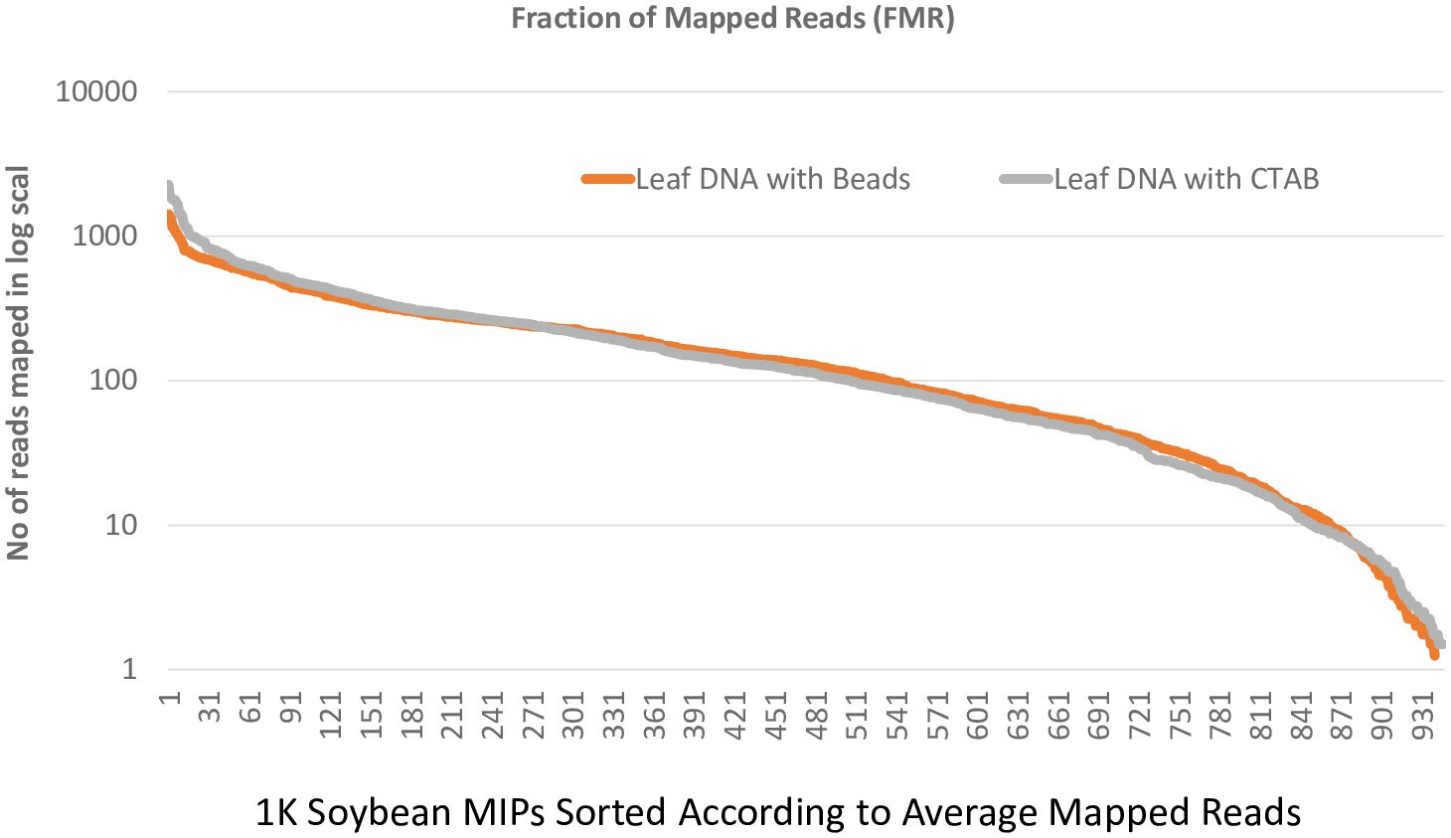
Performance of the molecular inversion probe (MIP) assay with isolated DNA. DNA samples isolated from soybean with SA-coated nanoparticles and CTAB method were run with the soybean 1K MIP marker set. From the 1K set 931 probes had one or more reads that aligned to their target sequence. The fraction of mapped reads were mapped for each probe. The probes are numbered 1–931 with probe one having the most reads mapped from the assay and probe 931 having the fewest reads mapped. Leaf DNA from beads and CTAB had similar number of reads mapped for each probe.

With DNA from soybean leaf, we further evaluated the quality of genomic DNA by using a targeted sequencing method. In total, 160,898–278,427 raw reads were obtained for the DNA samples prepared with beads+CTAB while the standard CTAB method produced 233,005–289,061 raw reads (S6 Table). After trimming and filtering low quality reads, in total, 948,597 reads from bead+CTAB (96% of total reads) and 1,063,223 reads from CTAB (96% of total reads) were kept (quality at > = Q 30). Both methods had a high percentage of reads mapped (S6 Table) with no significant difference between the samples from beads and CTAB method (p<0.05). As presented in form of Fraction of Mapped Reads (FMR) (Fig 4), with the 998 MIPs tested, at least one read was obtained for 964 of the MIPs for soybean leaf DNA prepared with beads while 973 MIPs had at least one read aligned in DNA sample extracted with standard CTAB method.

Moreover, the nearly identical FMR between two DNA samples (leaf DNAs from beads and CTAB) in MIPs further demonstrates that the quality of DNA prepared from these two methods is compatible for MIPs.

## Discussion

The role of magnetic nanoparticles in the DNA extraction process is to selectively adsorb and retain the target analytes (DNA in this study) from the sample matrix allowing them to serve as a solid surface for DNA. The magnetic properties of the nanoparticles allow for the analyte attached to the coated magnetic nanoparticles to be easily co-separated from the extraction material with a magnet. Specifically, this is very useful when the material for the analytes is a highly ‘contaminated complex’, such as the case with plant tissues being used for DNA extraction. Due to excessive contamination by secondary metabolites in plants, current plant DNA preparation methods need to be adjusted for their effectiveness and suitability for different plant/crop tissues [17–22] and for different molecular techniques [23, 24]. Among the methods, the CTAB method and its derivatives are still commonly used in plant DNA preparation [19, 25–27]. However, as stated before, these CTAB-based methods are not compatible with current environment and health safety concerns in the scientific and public community, because they inevitably result in the large amounts of waste of phenol and chloroform. Moreover, the additional manipulations involving phenol-chloroform can also reduce overall yield and may fail to produce quality DNA when the input material for DNA extraction is limited. Our results indicate that it is possible to isolate quality DNA directly after the lysis step with SA-coated nanoparticles from both leaf tissue and dry seed(s) in three different crops. Usually, the CTAB methods requires relatively large amount of lyophilized plant leaf tissues ≥ 0.01g and dry seeds ≥0.3 g for DNA preparation [28–30]. Using nanoparticles, the input materials for DNA extraction can go as low as 0.0034 g/5 discs as demonstrated with maize and wheat (S2 Table). Although the input material was low, we were able to isolate large amount of high-quality DNA. In addition, we noticed that as shown in Fig 2, the band of genomic DNA from soybean leaves was relatively not ‘sharp’, this might be related to the high proteins and polysaccharides attached to the DNA during isolation. But we saw no effect on the quality of the isolated DNA used in downstream analysis, such as PCR and MIP in this study. This allows the extractions to occur in 96 well deep well plates. Being able to process this extraction method in 96-well plates enables this protocol to be adapted to automation methods which can greatly increase throughput allowing for a thousands of samples to be isolated in a molecular breeding program. It is also possible to obtain higher yield of DNA using this method by adjusting the input volume of lysate. In this study a total of 500 μl of lysis buffer (CTAB buffer) was used in each extraction but only 100 μl of lysate was transferred into next step for DNA isolation with beads. By increasing the amount of lysate transferred, a higher amount of DNA could be obtained.

The successful application of DNA in both PCR (Fig 3) and MIPs (Fig 4) demonstrated the DNA is of acceptable quality for molecular techniques used in molecular breeding. In addition to quality, the labor needed and the cost to extract DNA are important factors when extracting a large number of samples for a molecular breeding program. With this protocol, 192 DNA isolations can be completed in less than <2 hours in 96 well plate format with fewer steps than a standard CTAB DNA extraction method. Overall cost, using SA-coated nanoparticles as DNA binding material can significantly reduce the cost per sample DNA. Only 120 ng of beads per 500 μl lysate (sample) was used per extraction which gives a cost of $8.77x10^-7^ beads per sample (Table 1). This is significantly lower than the cost when compared to other commercial bead extraction methods which can cost up to $23.87/ml.

## Conclusion

SA-coated nanoparticles can be used to isolate and quality-DNA from leaf tissues and seed of crops with other advantages,

- Magnetic nanoparticles can be easily synthesized in the lab.
- DNA can be isolated from both leaf tissues and seed(s) in less than <2 hours per preparation.
- The quality of DNA obtained with this method is suitable for downstream analysis, such as PCR and MIPs.
- The established method is fast, simple, reliable, and applicable for high throughput DNA preparation for crops.

## Supporting information

S1 Fig The paper puncher (Amazon, A7074005S) and relative size of leaf disc for DNA extraction. (PPTX)

S2 Fig Correlation of the number of leaf discs to the total yield of DNA in soybean, wheat and maize. (PDF)

S1 Table Primers used in amplification of targeted region in each crop. (XLSX)

S2 Table Primers used in sequencing on NextSeq500 Platform. (XLSX)

S3 Table Indexed primers used for individual samples for the MIP assay. (XLSX)

S4 Table Yield of DNA from seed(s) from three crops tested. (XLSX)

S5 Table Weight of leaf discs of three crops used in DNA isolation and yield of DNA extracted. (XLSX)

S6 Table Comparison of total reads obtained/filtered/aligned to reference in MIP analysis with DNA from soybean leaf. (XLSX)

S1 File Step-by-Step protocol for magnetic nanoparticle preparation, DNA extraction, PCR, PCR product evaluation and MIP assay, also available on protocols.io. (PDF)

S1 Raw images (PDF)
