# Supplementary figures and images for: Crop DNA extraction with lab-made magnetic nanoparticles
Source: PLoS One. 2024 Jan 8;19(1):e0296847. doi: 10.1371/journal.pone.0296847 (PMC10773960; doi:10.1371/journal.pone.0296847)

## Slide 1
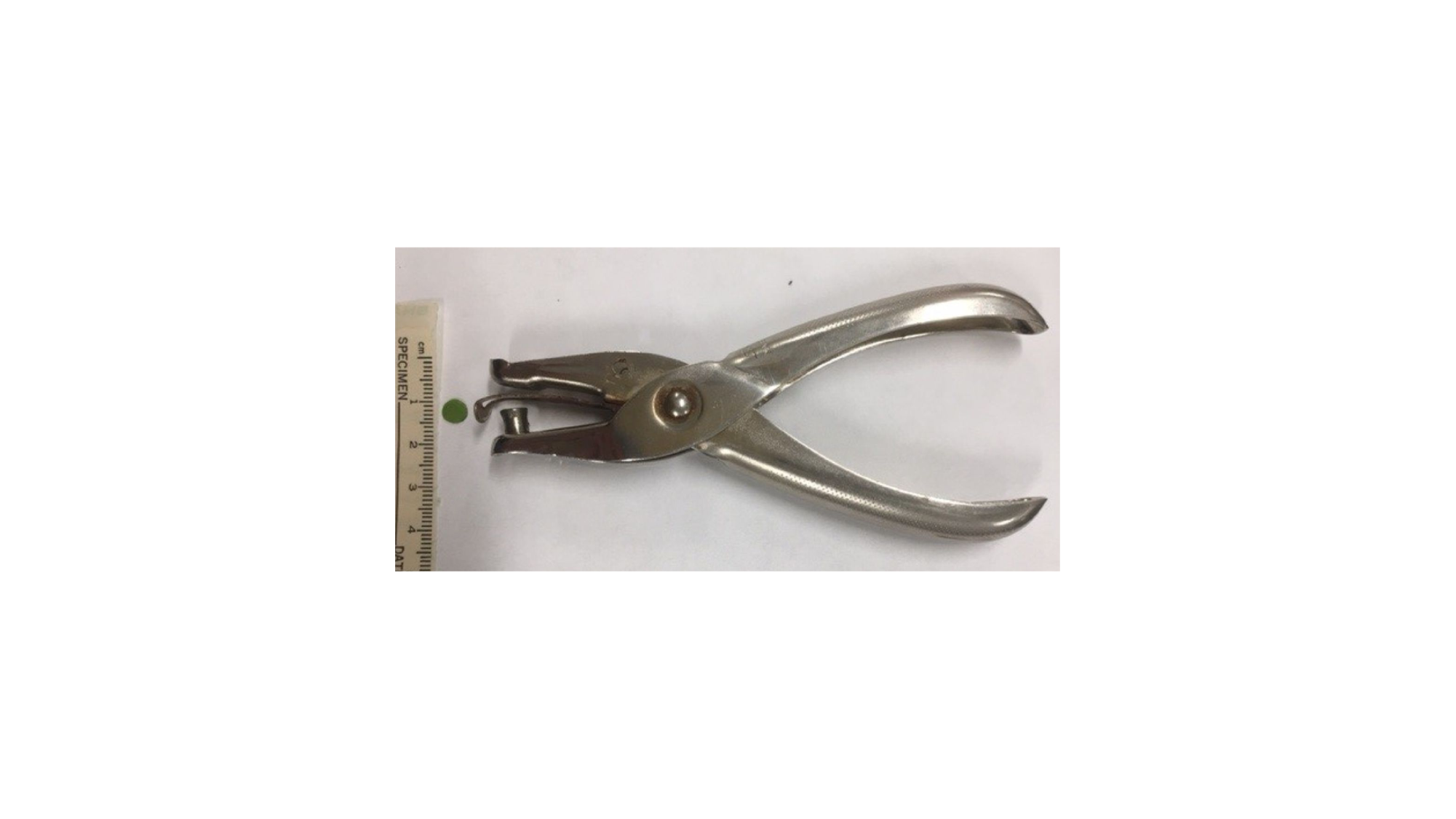

Supplement: S1 Fig — (PPTX) [file pone.0296847.s001.pptx]

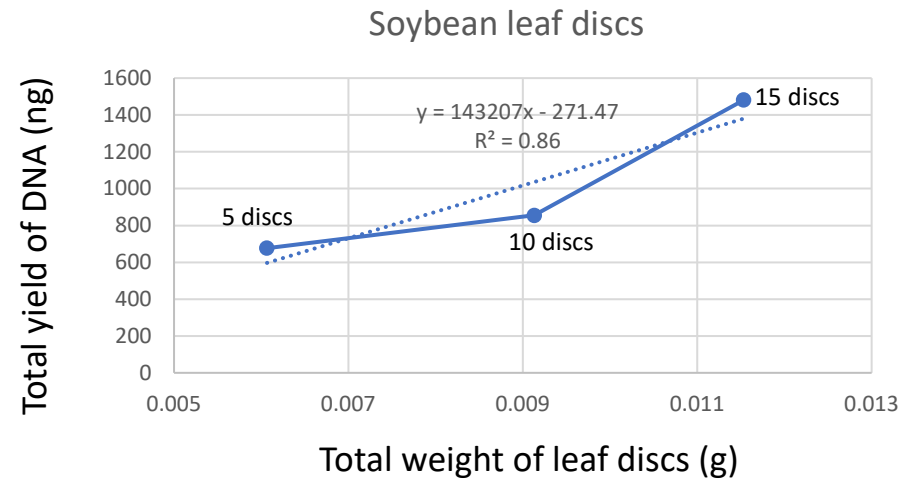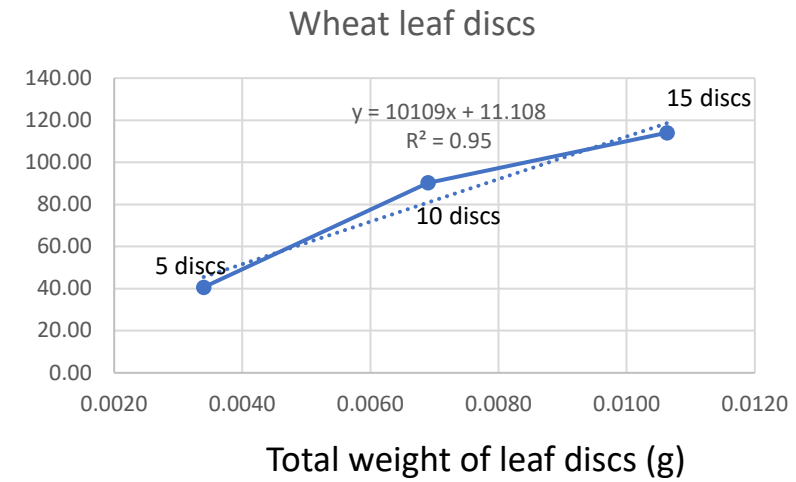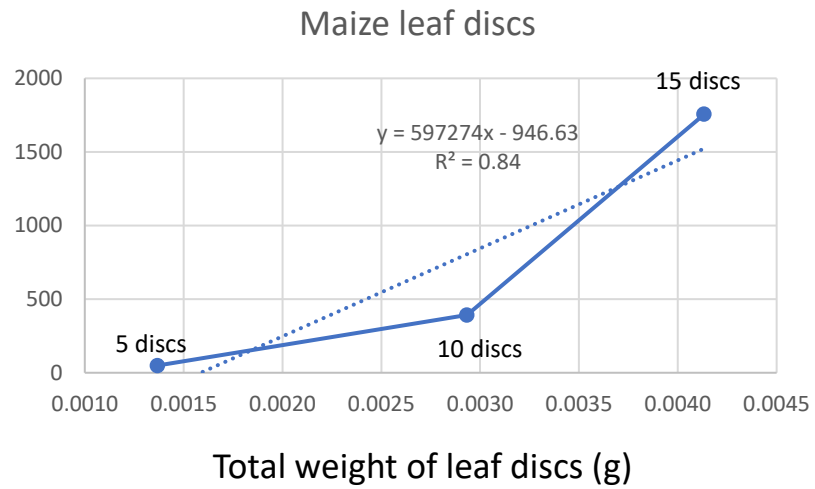

Supplement: S2 Fig — (PDF) [file pone.0296847.s002.pdf]
