# Supplementary material for: Crop DNA extraction with lab-made magnetic nanoparticles
Source: PLoS One. 2024 Jan 8;19(1):e0296847. doi: 10.1371/journal.pone.0296847 (PMC10773960; doi:10.1371/journal.pone.0296847)
Supplement: S1 File — (PDF) [file pone.0296847.s009.pdf]

Crop DNA Extraction with Lab-Made Magnetic Nanoparticles V.1

Haichuan Wang[1], Xueqi Zhao[2], Li Tan[3], Junwei Zhu[1], david.hyten[4]

[1]USDA-ARS; [2]School of Science, Xi'an Jiaotong University;
[3]Department of Mechanical & Materials Engineering, UNL, Lincoln NE 68588 USA;
[4]Department of Agronomy and Horticulture, UNL, Lincoln NE 68583 USA

USDA-ARS UNL soybean genomics

Haichuan Wang

VERSION 1
DEC 19, 2023

OPEN ACCESS

DOI:
dx.doi.org/10.17504/protocols.io.e6nvwdqm7lmk/v1

Protocol Citation: Haichuan Wang, Xueqi Zhao, Li Tan, Junwei Zhu, david.hyten 2023. Crop DNA Extraction with Lab-Made Magnetic Nanoparticles. **protocols.io** https://dx.doi.org/10.17504/protocols.io.e6nvwdqm7lmk/v1Version created by Haichuan Wang

License: This is an open access protocol distributed under the terms of the Creative Commons Attribution License, which permits unrestricted use, distribution, and reproduction in any medium, provided the original author and source are credited

ABSTRACT

Molecular breeding methods, such as marker-assisted selection and genomic selection, require high-throughput and cost-effective methods for isolating genomic DNA from plants, specifically from crop tissue or seed with high polysaccharides, lipids, and proteins. A quick and inexpensive high-throughput method for isolating genomic DNA from seed and leaf tissue from multiple crops was tested with a DNA isolation method that combines CTAB extraction buffer and lab-made SA-coated magnetic nanoparticles. This method is capable of isolating quality genomic DNA from leaf tissue and seeds in less than 2 hours with fewer steps than a standard CTAB extraction method. The yield of the genomic DNA was 582-729 ng per 5 leaf discs or 216-1869 ng per seed in soybean, 2.92-62.6 ng per 5 leaf discs or 78.9-219 ng per seed in wheat, and 30.9-35.4
ng per 5 leaf discs in maize. The isolated DNA was tested with multiple molecular breeding methods and was found to be of sufficient quality and quantity for PCR and targeted genotyping by sequencing methods such as molecular inversion probes (MIPs). The combination of SA-coated magnetic nanoparticles and CTAB extraction buffer is a fast, simple, and environmentally friendly, high-throughput method for both leaf tissues and seed(s) DNA preparation at low cost per sample.
The DNA obtained from this method can be deployed in applied breeding program for marker-assisted selection or genomic selection.

Protocol status: Working
We use this protocol and it's working

Created: Dec 19, 2023

Last Modified: Dec 19, 2023

PROTOCOL integer ID: 92516

Funders Acknowledgement:
Increasing the Rate of Genetic Gain for Yield in Soybean Breeding Programs

Nanopartcle synthesis, sample collection, DNA preparation, eval...

1. Weigh $FeCl_2 \cdot 4H_2O$ (4.34g, 0.0218 mol).

2. Weigh $FeCl_3 \cdot 6H_2O$ (10.84 g, 0.04 mol).

3. Mix both $FeCl_2 \cdot 4H_2O$ and $FeCl_3 \cdot 6H_2O$ in 94 ml deionized water well in a 500 ml glass flask. (the molar ratio of Fe(II) to Fe(III) is 0.55: 1).

4. Adequately filter the solution with filter paper (Whatman Cat. 1440-005) to remove impurities.

5. Add 50 ml NaOH (5.0 M) into the solution to obtain black magnetic nanoparticles (pH = 14) in a 500 ml three-necked, round-bottomed flask provided with a magnetic stirrer.

6. Heat at 60 °C for 30 minutes with vigorous stirring.

7. Immediately slow stirring for 1 h at 80 °C under a nitrogen atmosphere for a ferrofluid containing Fe_3O_4 nanoparticles.

8. Add 5g (0.08 mole) of SA powder into the suspension (~150 mL) of magnetic nanoparticles at a molar ratio of 0.55 Fe(II): 1 Fe(III): 2 SA.

9. Stir consistently at 90°C for 4 h.

10. Separate SA coated Fe_3O_4 from the aqueous solution by letting round-bottomed flask sit on regular magnet for 30 minutes at room temperature.

11. Remove aqueous solution by pouring.

12. Add 250 ml pure water into flask.

13. Stir on stirring station for 5 minutes.

14. Let flask sit on regular magnet for 30 minutes at room temperature.

15 Repeat step 11-14 five times.

16 Add 250 ml pure water.

17 Measure pH with pH meter (Thermo Scientific Orion Star™ A215 Benchtop pH/Conductivity Meter), a neutral pH (pH 7) should be reached.

18 If pH is not at 7, then repeat step 11-14 2-3 more times, until pH is at 7.

19 Keep the final solution with SA coated magnetic particles at room temperature in a sealed glass bottle (100 ml) for future application in DNA preparation.

20 Collect leaf discs in ¼ inch from soybean at V2 stage, wheat at Feekes 2 stage and maize at V3 stage by using a paper puncher (Amazon, A7074005S).

21 5, 10 and 15 leaf discs per collection per crop per tube (1.1 ml bullet tubes), three reps per crop.

22 Load the tubes on 2 ml Avan 96-well deep plate.

23 Add five 2 mm ceramic beads (Biospec. Cat. 11079124zx) into each tube.

24 Cover plate with cheese cloth and wrap with rubber band.

25 Turn on Virtis Freezemobile Lyophilizer (25x), temperature set at -80 °C and vacuum at 50 mT.

26 Load the plate with tubes on Lyophilizer (Virtis Freezemobile Lyophilizer (25x)) once the temperature and vacuum reach its setting.

27 Lyophilize leaf discs on Lyophilizer overnight at -80°C and 50 mT,

28 Remove the plate and seal tube with strip of caps for 1.2 ml micro titer tubes and store at room temperature for DNA extraction.

29 Collect 1, 2 and 3 dry wheat seed(s) and 1 dry soybean per 1.1 ml tube.

30 Add eight 2 mm ceramic beads (Biospec. Cat. 11079124zx) into each tube and seal tube with cap.

31 Add 500 µl extraction buffer into each tube with seed(s) and leave tubes in 4 °C fridge overnight before grinded.

32 Load the plate with tubes having leave tissues on the TissueLyserII (Qiagen) by using TissueLyser Adapter Set.

33 Shake for 30/s for 5 min.

34 Repeat step 24 one more time.

35 Remove tubes from tissuelyser.

36 Spin down at 3700 rpm for 30 sec.

37 Add 500 µl of DNA extraction buffer (Tris-HCl 50 mM, pH 6.5, CTAB 1%, EDTA 10 mM, beta-mercaptoethanol 5%, 1.4 M NaCl and 2% PVP-40) into each tube.

38 Seal tubes with strip of caps for 1.2 ml tubes well

39 Incubated plate with tubes having lyophlized leaf tissues at 65°C for 60 min on a water bath.

40 Clean the water on plate by using paper towel.

41 Centrifuge at 3700 rpm for 15 min at 25°C.

42 For grinding the seeds in tubes, follow the step 32-36 and step 38-41.

43 Take a new a new 0.2 ml 96-well non-skirt plate, add 2 µl beads (60 ng/µl) and 1 µl of RNase A (1 µg/µl) into each well bottom.

44 Transfer 100 µl supernatant per tube into well, one tube into one well.

45 Mix well by pipetting up and down 3 times and leave plate on bench for 10 min at room temperature.

46 Add 100 µl isopropanol into each well.

47 Mix well by pipetting up and down three times.

48 Leave plate on bench for 10 min at room temperature.

49 Let the plate sit on a magnet (dynaMag-96 side) for 5 min.

50 Remove all solution carefully from each well by pipetting. don't touch beads pellet on wall.

51 Add 100 µl 80% EtOH without disturbing the beads.

52 Wait for 30 sec.

53 Remove EtOH by pipette and air dry for 5 min.

54 Remove 96-well PCR plate from magnet.

55 Add 50 µl 10 mM Tris-HCl (pH 8).

56 Mix well with a pipette or slightly vortex.

57 Spin down the plate.

58 Leave plate on bench for 10 min at room temperature (not on magnet).

59 Place plate back on the magnet for 3-5 min.

60 Transfer solution (~ 50 µl) with DNA into a new PCR plate.

61 Leave the plate on ice.

62 Measure DNA concentration on Qubit (2.0) by following kit instructions (Qubit™ dsDNA Quantification Assay Kits, Q32850).

63 Load 1 µl of DNA/sample/well on 1.5% agarose gel with SYBR. Lambda DNA used as DNA marker.

64 Label the plate, seal plate with foil plate seal (Thermo Scientific, Cat.232698) and store plate at -20°C freezer for future use.

65 Run at 120 V for 45 min in 1x TBE buffer.

66 Evaluate the gel under UV Transilluminator (UVP) at 488 nm wavelength.

67 Create a daughter plate to a final concentration of 12 ng/µl/DNA sample for further evaluation by molecular inversion probe (MIP) and conventional PCR analysis, respectively.

68 Store the plate with all DNA samples in -20 °C freezer for use.

69 Mix 5 µl of 2x master mix (BioRad, Cat. 172-5310), 0.25 µl of 10 µM primers (Forward and Revers primer respectively) and 1 µl genomic DNA in a 200 µl PCR tubes (Axygen, Cat. PCR02C) at a final reaction volume of 10 µl/sample.

70 Conduct PCR with following conditions, 98 °C/30 s, 21x (98 °C/10 s, 60 °C/30 s, 72 °C/30 s), 72 °C/60 s, holding at 12 °C on BioRad C1000 thermal cycler.

71 Load 3 µl of DNA/sample/well on 1.5% agarose gel with SYBR along with 100 bp DNA ladder.

72 Run at 120 V for 45 min in 1x TBE buffer.

73 Evaluate the gel under UV Transillumnator at 488 nm wavelength.

Molecular Inversion Probe (MIP)

74 Switch to MIP protocol

Molecular Inversion Protocol (MIP)

75 Dilute 998 individual probe synthesized by Integrated DNA Technologies (Coralville, IA, USA) at 0.2 nmole (Supplemental Table S2, Wang et. al. 2022) with nuclease free water at 1:10.

76 Pool 2 µl each probe together in a 2 ml centrifuge tube.Phosphorylate the pooled probes in 200 µl PCR tube in a 20 µl reaction consists of 15 µl of diluted Pooled probes, 1× T4 DNA ligase buffer, 1 mM ATP, and 0.5 U of T4 polynucleotide kinase (ThermoFisher, T4 polynucleotide kinase kit).

77 Close the cap and mix well.

78 Spin down.

79 Load the PCR tube to thermal cycler and incubate at 37 °C for 30 min followed by an inactivation of the kinase at 75 °C for 10 min.

80 Spin down tube and leave tube on ice in an ice bucket. Make a master mix for hybridization and extension, which consists of 1.05 µl pooled phosphorylated

81 probes (~1,000:1 DNA to probe ratio), 1.25 µl 10× Ampligase DNA ligase buffer (Epicentre Technologies), 1 µl of 5 M Betain (Sigma Aldrich), 3.25 U AmpliTaq DNA polymerase (ThermoFisher), 16 U of DNA Ampligase (Epicentre Technologies), 0.016 mM dNTP, and water. Note, this is for one reaction/well. Scale it up accordingly with your sample size.

82 Take a new 384-well plate.

83 Add 5.25 µl at 12 ng/ul (63 ng) DNA sample into corresponding well. One sample/well.

84 Dispense the master mix from step 8 into each well by using Mantis Liquid Handler (Formulatrix). The final reaction volume is 12.5 µl per reaction per sample per well.

85 Seal the 384-well plate with film by using PCR Plate Sealer and mix well by vortexing.

86 Spin down.

87 Leave plate on ice.

88 Turn on the thermal cycler.

89 Load the plate to thermal cycler.

90 Run a program as following parameters. 98 °C for 10 min at 60 °C for ~24 h. Note, the heated lid for the PCR machine is set to 105 °C for the first 30 min and then changed to 80 °C after the first 30 min of the incubation.

91 Stop PCR reaction and remove the plate.

92 Spin down plate, then leave the plate on ice.

93 Remove the sealing film.

94 Dispense 2 µl mix of 6.4 U of Exo I (New England Biolabs), 32 U of Exo III (New England Biolabs), 0.2 µl 1× Ampligase DNA ligase buffer into each well using a Mantis Liquid Handler (Formulatrix).

95 Seal plate with seal film using PCR Plate Sealer, mix plate well by vortexing and spin down.

96 Load the plate to thermal cycler and incubate at 37 °C for 30 min followed by 95 °C for 2 min to digest uncircularized MIPs and DNA templates.

97 Remove the plate from thermal cycler, spin down and leave it on ice.

98 Make a PCR master mix 2.5 µl of 2× iProof HF Master Mix (BioRad) and 0.75 µl water in a 200 µl PCR reaction tube. (Note, this volume is for one reaction per well, you need to

scale up volume accordingly with your sample size).

99 Close the cap mix well by vortexing.

100 Spin down and leave tube on ice.

101 Take a new 384-well PCR plate.

102 Add 0.5 µl of 10 µM indexed forward and reverse primers (Supplemental Table S2) per well.

103 Transfer 1.25 µl of circularized MIP capture taken from step 24 as template by using multichannel pipette.

104 Dispense 3.75 µl master mix from step 27 into each well by using Mantis.

105 Seal plate with seal film, vortex slightly to mix well.

106 Spin down the plate.

107 Load the plate to the thermal cycler.

108 Run PCR by following conditions: 98 °C for 30 s; 21 cycles of 98 °C for 10 s, 60 °C for 30 s, 72 °C for 30 s;and 72 °C for 60 s, holding at 12 °C.

109 Spin down the plate.

110 Remove the seal film.

111 Pool PCR reactions by taking 1 µl/well/sample in a 1.5 ml centrifuge tube.

112 Mix well and spin down, then leave tube on ice.

113 Take 30 µl pooled PCR products from step 39 and separate it on 1.5% agarose gel along with 50-bp (Fisher) and 100-bp DNA ladders (GoldBio) for 25-30 min at 120 V.

114 Stain the gel with 1x SYBR in running buffer for 5-10 min.

115 Excise the sharp band at ~286 bp by using clean razor blade.

116 Purify gel from step 42 with gel extraction kit (Qiagen) by following the manufacturer's instructions.

117 Quantify the purified PCR products on Qubit (ThermoFisher) with Qubit DNA HS Assay Kit (ThermoFisher).

118 Quantify purified pooled PCR products further precisely by using the KAPA library quantification kit Illumina platforms (KAPA, KK4824) on a QuantStudio 6 Flex system (Thermo Fisher Scientific).

119 Sequence the purified PCR products on NextSeq 550/500 system (Illumina) at 150 bp single read with customer specific sequencing primers (Supplementary Table S3).

120 Filter the raw reads, including remove adapter sequence contamination, truncated reads, and reads with overall low base quality the raw reads with Trimmomatic (software).

121 Aligned the filtered reads to a custom reference genome, which consists of 200-bp flanking regions around each targeted SNP for each of the 998 SNPs using Bowtie 2 in end-to-end mode with the sensitive setting.

122 Discard reads with a mapping quality score of 20.
