# Supplementary material for: Crop DNA extraction with lab-made magnetic nanoparticles
Source: PLoS One. 2024 Jan 8;19(1):e0296847. doi: 10.1371/journal.pone.0296847 (PMC10773960; doi:10.1371/journal.pone.0296847)
Supplement: S1 Raw images — (PDF) [file pone.0296847.s010.pdf]

Figure 2 images

Soybean seed DNA A1-H1

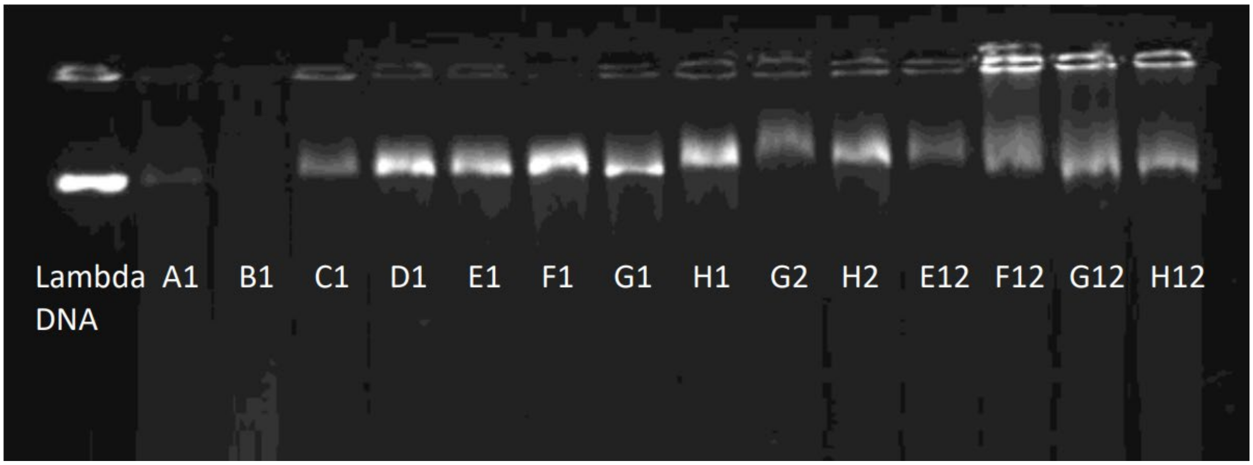

Leaf DNA gel

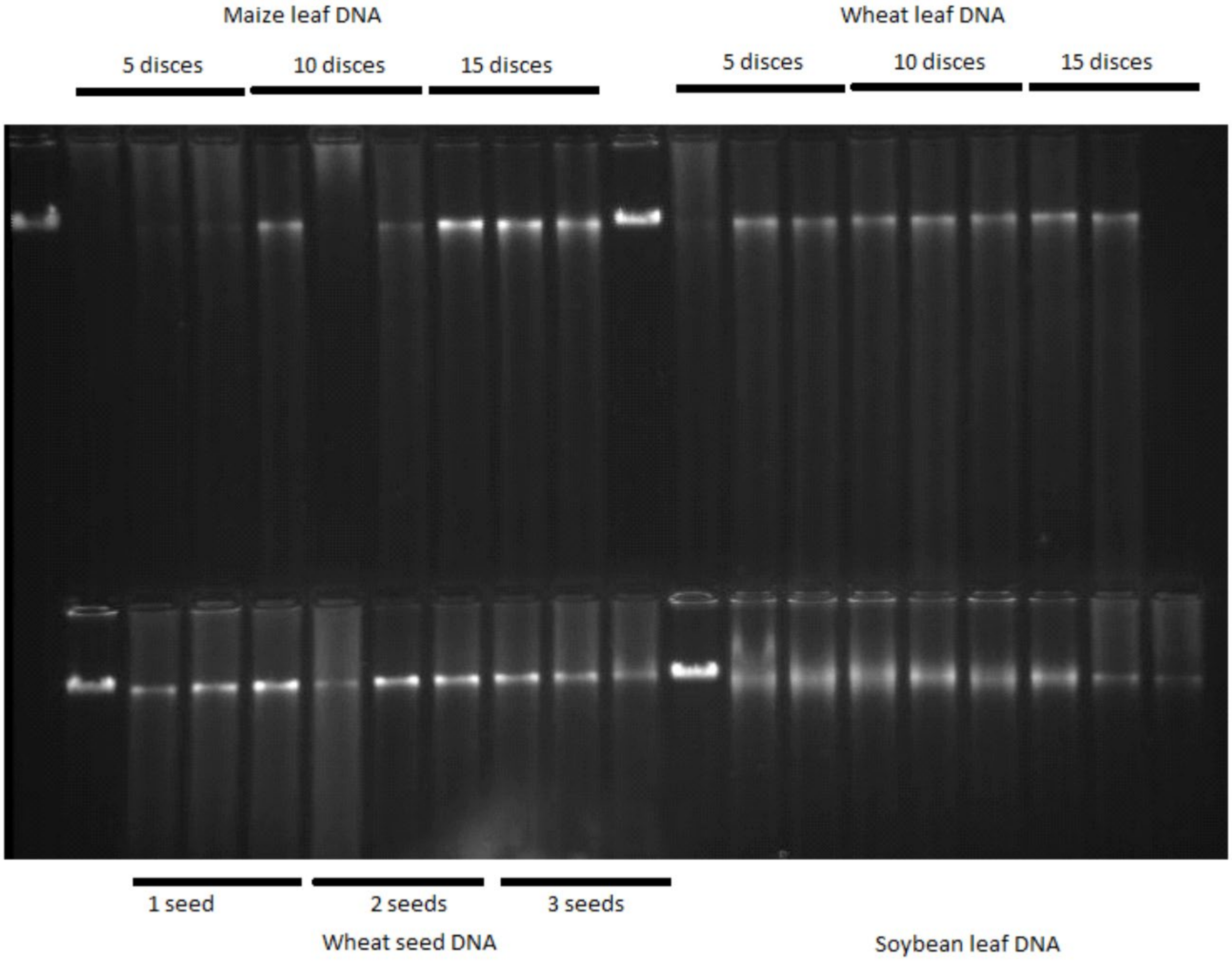

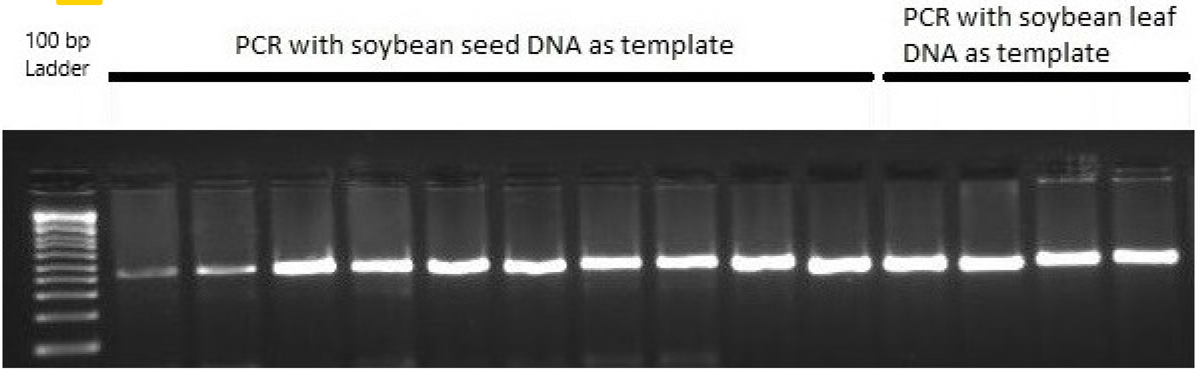
100 bp Ladder
PCR with soybean seed DNA as template
PCR with soybean leaf DNA as template

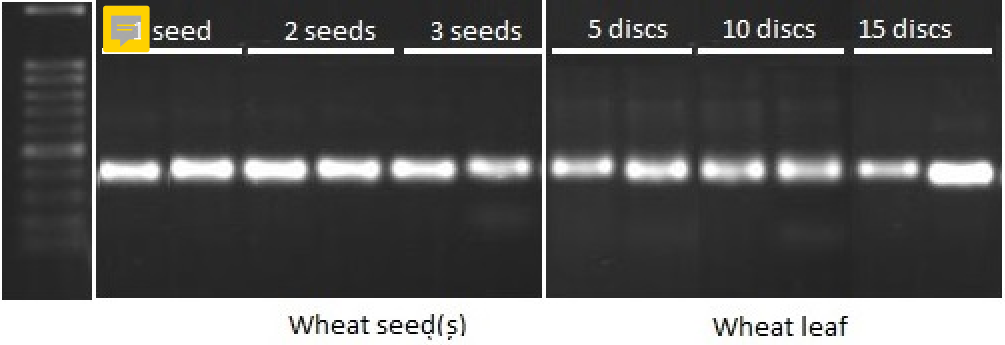
1 seed
2 seeds
3 seeds
5 discs
10 discs
15 discs
Wheat seed(s)
Wheat leaf
